# Social Anxiety and Pro-social Behavior Following Varying Degrees of Rejection: Piloting a New Experimental Paradigm

**DOI:** 10.3389/fpsyg.2019.01325

**Published:** 2019-06-21

**Authors:** Joanneke Weerdmeester, Wolf-Gero Lange

**Affiliations:** Behavioural Science Institute, Radboud University Nijmegen, Nijmegen, Netherlands

**Keywords:** social anxiety, social exclusion, rejection, pro-social behavior, degree of rejection, social reward, approach-avoidance

## Abstract

In general, human beings tend to try and reconnect after they have been socially rejected. It is not clear, however, which role the number of rejecters and rejection sensitivity plays. In addition, it is unclear whether the supposed pro-social behaviors are aimed at the rejecters or at innocent individuals. By means of a new paradigm, the present pilot study investigated compensatory behavior of individuals with varying degrees of social anxiety, following varying degrees of rejection. In addition, it was explored toward whom their behavior was directed: rejecters or innocent individuals. Female students (*N* = 34) were assessed on their degree of social anxiety and then, based on a personal profile they wrote, they were either rejected by 1, 2, or 3 fictional other participants or completely accepted. Afterward, the participants had to explicitly rate the creativity of drawings made by the others and, in a pro-social reward paradigm, awarded the other participants money based on their creativity rating. In addition, implicit social approach tendencies toward photos of rejecters, acceptors, or innocent individuals were assessed by means of an approach-avoidance task. The results confirmed that people with a low degree of social anxiety respond to rejection in a compensatory pro-social manner explicitly as well as implicitly, but that people with a high degree of social anxiety fail to do so. With regard to sources of rejection, only implicit approach-avoidance tendencies reflected a distinction between rejecters and innocent individuals. Theoretical implications are discussed in the light of the small sample size and other limitations.

## Introduction

Human beings have an evolutionarily determined (DeWall, 2013) “need to belong” which may have ensured survival in prehistoric times and still underlies a great part of our social behavior today (Baumeister and Leary, 1995). Yet, there was also a strong need to be selective in the formation of social bonds in order to avoid those reducing fitness and to engage in bonds leading to fitness benefits (Kurzban and Leary, 2001; Wesselmann and Williams, 2017). As exclusion from a group significantly jeopardized survival in our ancient past, a breach to one’s need to belong has evolved to be, and still is accompanied by potent negative feelings of anxiety, depression, loneliness, and even self-dehumanization (Baumeister and Leary, 1995; Williams and Nida, 2011; Bastian et al., 2013). Accordingly, this leads to a pervasive drive of human beings to maintain existing and re-establish broken relationships (belongingness hypothesis). Therefore it is frequently hypothesized that social exclusion leads to compensatory pro-social behavior in order to regain acceptance. Research has shown that individuals after being ostracized worked harder on a collective group task (Williams and Sommer, 1997), were more likely to conform to the opinion of others (Williams et al., 2000), and were more likely to comply with social influence tactics (Carter-Sowell et al., 2008). Furthermore, it was found that, after ostracism, participants showed increased interest in new groups (Maner et al., 2007), when compared with included participants. In addition, they showed increased attention to affiliative facial expression (Xu et al., 2015; Chen et al., 2017) and were more likely to mimic the body language of interaction partners or facial expressions intending to increase rapport (Lakin and Chartrand, 2005; Lakin et al., 2008; Cheung et al., 2015).

As opposed to frequently used ostracism paradigms, in which individuals are merely ignored while, e.g., participating in a multi-player online ball-tossing game (Williams et al., 2000; Hartgerink et al., 2015), Mallott et al. (2009) rejected participants in their study more explicitly. Participants recorded a video message, which was supposedly shown to others. Based on this message, they were either rejected or accepted later on. Results showed that rejected participants rewarded drawings made by others with more money, than accepted participants. Twenge et al. (2001), on the other hand, showed that rejected participants evaluated others more negatively and gave them more hot sauce to eat (which is seen as index for aggressive behavior, Ayduk et al., 2008) in comparison to non-rejected individuals. In a study by Buckley et al. (2004), participants were gradually rejected or accepted by another ostensible participant, based on a self-written personal profile. Here, rejected participants selected more unpleasant audiotapes for the rejecter to listen to (for comparable results see Twenge et al., 2001; Warburton et al., 2006). In a series of studies, Twenge and Campbell (2003) investigated the influence of narcissism and self-esteem on rejection responsivity. They rejected participants by means of either instructed recollection of a rejection situation or actual rejection from a group by “peer vote.” Their results showed that increases in self-reported anger and increased “punishment” of former rejecters by means of louder and longer blasts of “white noise” (Taylor Aggression Paradigm; Taylor, 1967) were related to degrees of narcissism but not self-esteem. Based on these contradictory results, it has been proposed that individual and situational factors may contribute to how someone responds to exclusion. For example, it has been suggested that an increasing degree of rejection is related to an accumulation of negative affect (Williams et al., 2000). However, to our knowledge, the association with frequency or intensity of rejection on resulting behavior has only been explored infrequently and in no systematic way so far. For example, Sandstrom et al. (2017) reported that children become increasingly distressed by an increasing number of rejecters. In the same line, Tobin et al. (2018) reported increased sadness and anger in people ostracized by larger as compared to smaller groups. On the other hand, Pfeiffer and In-Albon (2016) found no such effects in adolescents. It is also unknown, whether affiliative behavior following rejection is exclusively aimed at the perpetrators of rejection to reconcile or at others in general to re-affiliate as quickly as possible.

With regard to individual factors, it seems that high rejection sensitive individuals, when compared to low rejection sensitive individuals, evaluated their interaction partners less positively after rejection (Ayduk et al., 1999) and gave them more hot sauce to eat (Ayduk et al., 2008). The core fear of individuals with high degrees of social anxiety (HSAs) and patients with social anxiety disorder (SAD) is to be negatively evaluated by others and to be eventually rejected (e.g., Rapee and Heimberg, 1997; American Psychiatric Association, 2013). Although fears of negative evaluation seem to be normally distributed in the population (Rapee and Heimberg, 1997), it appears that individuals at the higher end of this distribution are not only more tense in social interactions but also increasingly avoidant of social situations. To fulfill the criteria of a *diagnosis* of SAD, these same fears and avoidance behaviors must lead to significant disruptions of social, educational, or professional functioning (American Psychiatric Association, 2013). Based on this increased fear of rejection, this group has been heavily investigated in ostracism and rejection research in the last years. If assuming that the fear of rejection is particularly strong in HSAs, one would expect that they would show even more re-affiliative behavior after rejection. Yet, potential rejection stresses them to such a high degree that it seems to undermine pro-social behavior, instead. It has, e.g., been found that after being ostracized in a Cyberball game, HSAs have shown prolonged negative emotions as well as prolonged impairments in self-regulation (Oaten et al., 2008). In the same line, Heeren et al. (2017) investigated brain activation in SAD patients and control participants after ostracism and suggested that SAD may be characterized by a “poor ability to recover” (Heeren et al., 2017, p. 1) *after* rejection rather than particular differences *during* the social exclusion. Breen and Kashdan (2011) found that participants’ social anxiety predicted feelings of anger after imagined rejection. In Mallott et al. (2009), HSAs showed the signs of avoidance such as decreased eye contact after rejection and giving others less reward than non-anxious controls. In fact, these findings are in line with cognitive theories Clark and Wells, 1995; Rapee and Heimberg, 1997) as well as with a theoretical framework suggesting that HSAs tend to preferentially process belief-confirming above belief-disconfirming information (Belief-bias; Vroling et al., 2016). This implies that HSAs would experience explicit or putative rejection as confirmation of their initial beliefs and fears, strengthening them even further, and deem any action to countervail the rejection pointless (Clark and McManus, 2002; Hofmann and DiBartolo, 2010). Congruently, only few studies could evidence that people fearing rejection would show increases in affiliative behavior after rejection (e.g., Romero-Canyas et al., 2005, 2010; Williams et al., 2005; Williams, 2007, 2012; Leary and Hoyle, 2009).

In sum, most studies seem to indicate that rejection-sensitive individuals such as HSAs fail to show re-affiliative behavior after rejection (in contrast to normal controls), thereby contributing to a self-fulfilling prophecy. Yet, the circumstances and directions under which either re-affiliation or social withdrawal, anger, or retaliation occurs are not investigated in a systematic way.

In the current pilot study, inspired by Buckley et al. (2004) and Mallott et al. (2009), we propose an experimental procedure to systematically investigate the impact of degree of rejection and the direction of re-affiliation on explicit as well as implicit compensatory pro-social behaviors and provide first evidence for its workability. As social anxiety is more commonly observed in women than it is in men (Fehm et al., 2005; Asher et al., 2017), females with varying degrees of social anxiety were rejected by zero, one, two, or three others, based on feigned evaluations of their personal profile (Buckley et al., 2004). Then, participants explicitly evaluated and rewarded the creativity of drawings their rejecters and/or accepters had made (Mallott et al., 2009). To measure implicit social approach-avoidance tendencies an approach-avoidance task (AAT; Voncken et al., 2012) was used. Here, the participants saw the photos of rejecters, accepters, and unknown individuals in profile on a computer monitor. Depending on the instructions, participants either pulled or pushed a computer joystick. On pulling, the individual on the photos gradually turned to face the participants (approach), while pushing the joystick made the individual in the picture turn away (avoid).

Based on the belongingness hypothesis (Baumeister and Leary, 1995; DeWall, 2013) and evidence that human beings engage in compensatory pro-social acts after rejection (Williams and Sommer, 1997; Williams et al., 2000; Lakin and Chartrand, 2005; Maner et al., 2007; Carter-Sowell et al., 2008; Lakin et al., 2008; Mallott et al., 2009), it was expected that rejection (compared to acceptance) would lead to increased rewarding of others as well as increased approach tendencies toward face pictures. Additionally, we expected that reward behavior and approach tendencies would accumulate with an increasing number of rejecters (Williams et al., 2000). Furthermore, in line with Vroling et al. (2016), it was expected that with increasing levels of social anxiety, the reward given by rejected individuals would decrease and would lead to a stronger avoidance tendency (Ayduk et al., 1999; Ayduk et al., 2008; Oaten et al., 2008; Mallott et al., 2009; Breen and Kashdan, 2011). Due to overgeneralization of negative experiences, it was finally expected that with increasing degrees of social anxiety, the differentiation of rewarding and avoidance between rejecters and accepters (innocent individuals) would diminish (Maner et al., 2007; Mallott et al., 2009; DeWall, 2013).

Exploring the workability of experimental paradigms to systematically study the role of social anxiety in response to social rejection is particularly important when considering that HSAs seem to show subtle affiliative deficits in social interactions (Lange et al., 2014) and are more likely to generalize from one experience of (putative) exclusion in an interaction, to future interactions, thereby missing opportunities for affiliation with new partners (DeWall, 2013).

## Materials and Methods

### Participants

In order to explore the workability of the proposed setup, the sample in this pilot study consisted of 34 female students and graduates from Radboud University and the University of Applied Sciences (HAN) in Nijmegen, the Netherlands (Table 1), in the age range of 18–29 (*M =* 21.29, SD *=* 3.09). Participants were recruited through an online research participant system (SONA) of the Behavioural Science Institute, Nijmegen. The study consisted of one 30-min session and a 60-min session. Participants received either 1.5 credit points or 15 euros in gift certificates.

**Table 1:** Baseline characteristics of participants allocated to varying rejection conditions.

Number of rejecters					
Characteristic	Zero (*n* = 9)	One (*n* = 8)	Two (*n* = 9)	Three (*n* = 8)	*F*/*χ*^2^
Age	20.56 (2.65)	22.25 (2.82)	22.22 (2.95)	20.13 (3.83)	*F* = 1.09	
Social anxiety	24.44 (8.08)	32.00 (6.72)	32.00 (8.34)	28.25 (6.36)	*F* = 2.05	
Mother tongue					*χ*^2^ = 0.29
Dutch	9 (100%)	8 (100%)	7 (77%)	7 (87%)	
German	0 (0%)	0 (0%)	2 (22%)	1 (12%)	
Education					*χ*^2^ = 0.19
Psychology	3 (33%)	2 (25%)	3 (33%)	5 (62%)	
Pedagogy	3 (33%)	2 (25%)	1 (11%)	2 (25%)	
Other	1 (11%)	4 (44%)	3 (33%)	1 (11%)
Graduated	2 (22%)	0 (0%)	2 (22%)	0 (0%)	

Table represents numbers (percentage) or means (standard deviations) for each variable.

### Material and Procedure

#### First Session

**Figure 1:**
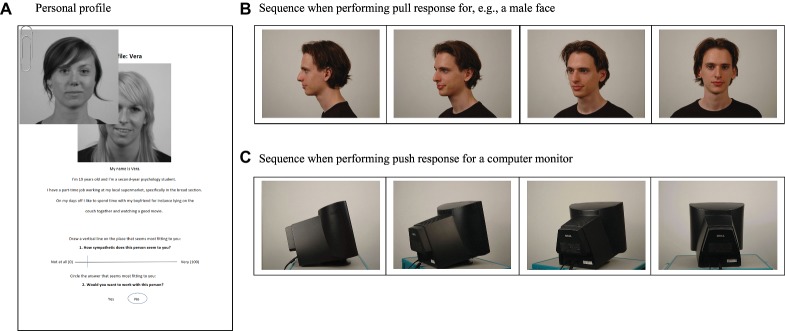
Examples of rejected personal profile with photo of ‘rejecter’ on top **(A)**, and, adapted from Voncken et al. (2012) with permission of SpringerNature: AAT stimuli when pulled **(B)** or when pushed **(C)**

Participants were informed about the procedure of the entire experiment and signed a consent form. Then they fill out online questionnaires through the online survey platform Unipark EFS (Globalpark, 2011) assessing socio-demographics. These included current age, their field of study, and mother language. In addition, this questionnaire included an assessment of their degree of social anxiety (for overview, see Table 1). A core aspect of social anxiety was assessed by means of the *Brief Fear of Negative Evaluation Scale* (BFNE; Leary, 1983), measuring the fear of being negatively evaluated by others. This scale has excellent psychometric properties (Collins et al., 2005). For 12 items (e.g., “*I am frequently afraid of other people noticing my shortcomings*”), participants indicated on a five-point Likert scale how much a statement applied to them (1 = “not at all characteristic of me” to 5 = “extremely characteristic of me”). Subsequently, participants wrote four short sentences describing themselves and were then photographed. They were placed on a swivelling office chair in front of a gray wall and eight pictures were taken, turning the chair in steps of 30° to mimic the stimuli in the Face-Turn Approach-Avoidance task (AAT; Voncken et al., 2012). Participants were informed that this information would be used to construct a personal profile, which they would exchange with other participants in the second session before meeting them in person. They were also told that the pictures would be used in a computer task (but that pictures would be deleted after participation). After this first session, the researcher constructed a profile page of the participant, using the four sentences and a photograph of the participant facing straight into the camera (Figure 1A). Three other profiles were also constructed to represent the fictional individuals the participant would supposedly interact with. Photographs were derived from the Radboud Emotional Face Database (RaFD; Langner et al., 2010) and were included in the AAT (Voncken et al., 2012). The second session was planned 1 or 2 days later.

#### Second Session

Participants were handed the three profiles of the fictional other individuals. They were instructed to read the profiles and to answer two questions: First, they had to indicate how sympathetic this person seemed to them on a visual analogue scale (VAS; Torrance et al., 2001), a 100 mm horizontal line ranging from 0 = “not at all” to 100 = “very much.” Second, they were asked: “Would you like to work with this person?”, which could be answered by circling “yes” or “no.” Afterward, they completed four additional VASs allowing us to assess mood changes due to the inclusion/exclusion manipulation: “How positive do you feel right now?”; “How anxious do you feel right now?”; “How angry do you feel right now?”; and “How much would you like to escape from this situation?”, again ranging from 0 = “not at all” to 100 = “very much”. VAS scales have been shown to accurately and validly measure state affect (McCormack et al., 1988), and, in this particular case, served as a manipulation check. After giving the instructions, the researcher left the room, supposedly to visit the other participants. After several minutes, the researcher returned and collected the rated profiles and the VAS scales. They told the participant that they would leave, again, to inform the others how the participant had rated their profiles and would return with the others’ evaluation of her profile. Meanwhile, the participants were instructed to draw a building for later evaluation.

Outside the lab, the researcher prepared the ratings of the participant’s profile by the “others” according to the randomly assigned conditions (number of rejecters): For the question “Would you like to work with this person?”, “No” was circled on either one, two, all three, or none of the copies. Accordingly, the VASs concerning the evaluation of sympathy of the participant would be marked at 10 or 20 mm in case of rejection (close to “not at all” sympathetic), and at 80 or 90 mm in case of acceptance (close to “very much”). Subsequently, three stacks were prepared with in the upper-left corner a photograph of the person that had supposedly rated the profiles, followed by the rated profile of the participant, and the participant’s profile rated by the two other fictional individuals (Figure 1A). Consequently, this resulted in every participant receiving three of their profile copies back, each marked in a way that each participant is at randomly rejected (i.e., “no” circled in reply to question “Would you want to work with that person?” with respective sympathy ratings) by either none, one, two, or all three of the imaginative other participants. The assignment of the photographs/fictional participants to a certain role (accepter or rejecter) was counterbalanced.

The researcher and participant would then go through the rated profiles together. In the rejection conditions, the researcher would explain that the meeting with the others would be cancelled as it would be unethical to force participants to work together although they do not want to. In the acceptance condition, they were told the same, but now because one of the other participants was indicated as someone that the others did not want to work with. In all instances, it was explained that this was not foreseen and that, as a consequence, the planned interaction task had to be omitted.

The researcher then explained that they would proceed with a task that had been planned to follow the interaction task. For this reward assignment task (Mallott et al., 2009), participants were, in a rigged lottery, assigned to judge the drawings of the other participants in an art contest, rather than being contestant. While the researcher would pick up the drawings supposedly made by the other participants, the participant was asked to fill in the four VASs related to their current affect state, again.

After their return, the researcher spread out the drawings in front of the participant with the photos of the contestants visible. Again, the assignment of these drawings to one of the three individuals/photos was counterbalanced. The researcher placed an evaluation form beside the participant, on which they wrote down the names of the contestants, and assigned them with a letter (A, B, or C). To measure explicit pro-social behavior, participants were instructed to judge each drawing on creativity (0 = “not at all creative” to 100 = “very creative”) and to reward the contestants by distributing money (10 euro in coins, provided by the researcher) over four different cups, labeled with A, B, C, or “raffle.” They were informed that money placed in the raffle cup would be gathered throughout the whole study and then raffled among participants. Then, the researcher left the room while the participant rated the pictures.

Upon finishing the reward task, the researcher set up and explained the Face-Turn AAT task (Figures 1B,C; for technical details, see Voncken et al., 2012). Participants were instructed to either pull a joystick when faces or computer monitors on the screen were, e.g., facing left and push when facing right. Every type of stimulus [8 monitors, 8 male, 8 female (among which the 3 “fictional characters”)] was shown twice in each of 10 blocks, once facing left and once facing right. Participants were first given one practice trial for each type of stimulus (monitor, female and male) and direction. Subsequently, they performed the 10 blocks of 48 pre-randomized trials. Participants were given a short break after 120 trials and again after 240 trials. Then, the instructions were reversed (e.g., pull left and push right). They were again given the opportunity to practice, before finishing the last 240 trials (with a short break after 120 trials). The order of instructions was counterbalanced across participants. The reaction time and accuracy of the responses were recorded. Pictures of the three fictional participants were used to assess responses directed toward specific rejecters as well as innocent individuals. Specifically, 60 of the 480 trials/stimuli showed the fictional participants while 420 depicted unfamiliar stimuli (160 male, 160 monitors, and 100 females). Finally, participants were compensated, debriefed, and thanked.

### Data Analysis

A bootstrap re-sampling procedure (1,000 iterations, 95% confidence interval) was used to compensate for unequal sample sizes between some of the compared groups. Participants’ explicit behavioral responses to (degree of) rejection, in relation with their levels of social anxiety, was analyzed by means of an ANCOVA with money given to others as the dependent variable (DV), condition [acceptance vs. (degree of) rejection] as between-subject independent variable (IV) and social anxiety (total score BFNE) as covariate. Further, to be able to explore behavioral differences of a participant directed at accepters vs. rejecters, a subset of those participants was analyzed who were accepted and rejected by at least one person. This was done by using a repeated-measures ANCOVA, with target (mean amount of money given to rejecters versus innocent individuals) as within-subject factor and social anxiety as covariate.

To analyze implicit responses toward (degree of) rejection in relation with their varying levels of social anxiety, first, AAT scores were calculated, by subtracting the pull response time (RT) scores from the push RTs for the same stimuli. Computer monitors were seen as filler material and responses to them were not analyzed. An ANCOVA was performed with AAT score as DV, condition as the between-subject IV and social anxiety as covariate. Lastly, the difference in participants’ implicit responses toward new or innocent individuals compared to their rejecters was analyzed with a repeated-measures ANCOVA with target (AAT response toward rejecters and innocent individuals) as within-subject variable and social anxiety as covariate. Again, only the data from participants who were rejected by either one or two individuals were used.

As manipulation check, repeated-measures analyses were performed to investigate changes in subjective mood from before to after the manipulation. Reported scores for positive mood, anger, desire to escape, and anxiety were added as DVs, with time (before vs. after the manipulation) as within-subject variable and condition (acceptance vs. rejection) as between-subject variable. Subsequently, the analyses were repeated with degree of rejection as between-subject variable instead.

Several scatterplots were made to provide additional clarification of the results. Where necessary, additional *post hoc* (Pearson bivariate correlation) analyses were performed to examine significant or trending interactions with social anxiety, mood, and/or conditions. In the light of the small sample sizes of the subsamples, it has to be stressed that these follow-up analyses were merely of an exploratory nature.

## Results

### Baseline Differences

Participants rejected by either zero, one, two, or three individuals did not differ statistically, in terms of age, nationality, education, or social anxiety (Table 1).

### Pro-social Reward Paradigm

#### Rejection vs. Acceptance

**Figure 2:**
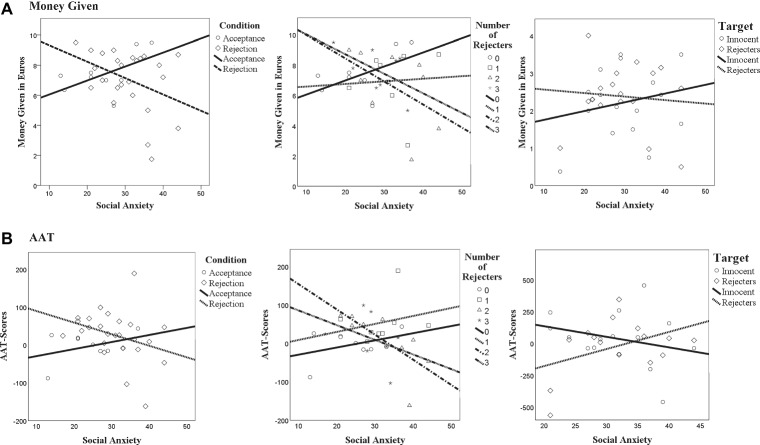
Scatterplot showing the relationship between condition, social anxiety and money given **(A)** or approach-avoidance responses **(B)**.

Results revealed a non-significant trend of condition, *F*(1, 30) = 4.39, *p* = 0.05, $\eta_{p}^{2}$ = 0.13, with rejected participants (*n* = 25) given less money in comparison to accepted participants (*n* = 9; see Table 2). This main effect was qualified by a significant interaction between social anxiety and condition, *F*(1, 30) = 5.84, *p* = 0.04, $\eta_{p}^{2}$ = 0.14 (Table 2). Explorative follow-up Pearson bivariate correlation analysis suggested a non-significant trend toward a negative relationship between social anxiety and the amount of money given in the rejection condition (*n* = 25), *r* = −0.39, *p* = 0.06 (two-tailed), which may mean that the higher someone’s degree of social anxiety was, the less money they gave. A visual inspection of the interaction effect (Figure 2) supported that notion. In the acceptance condition (*n* = 9), social anxiety was not correlated to amount of money given, *r* = 0.56, *p* = 0.12 (Figure 2A, left), while visual inspection suggests an *increase* in money given when one’s degree of social anxiety is higher.

**Table 2:** Money given to others and approach-avoidance scores across conditions.

	Money given		AAT scores	
Condition	*M*	SD	*M*	SD
Acceptance (*n* = 9)	7.39	1.34	−2.90	37.85
Rejection (*n* = 25)	7.06	2.01	25.72	66.90
**Number of rejecters**				
Zero (*n* = 9)	7.39	1.34	−2.90	37.85
One (*n* = 8)	6.96	2.00	54.39	57.45
Two (*n* = 9)	6.62	2.48	9.32	76.13
Three (*n* = 8)	7.66	1.49	15.52	63.37
**Target**				
Innocents (*n* = 17)	2.37	0.92	36.28	210.90
Rejecters (*n* = 17)	2.24	0.85	−21.73	219.68

AAT scores, Approach-avoidance task scores, calculated as: push response times-pull response times for the same stimuli.

#### Number of Rejecters

Results revealed no significant difference in amount of money given when someone was rejected by either zero, one, two, or three individuals, *F*(1, 26) = 1.78, *p* = 0.18, $\eta_{p}^{2}$ = 0.17 (Table 2). In addition, there was no significant interaction between social anxiety and the number of rejecters, *F*(1, 26) = 2.01, *p* = 0.14, $\eta_{p}^{2}$ = 0.19. Visual inspection of the interaction between social anxiety and the number of rejecters generally supports the results from the previous analysis section: A general decrease of money given, when social anxiety increases; however, this mostly seems to be the case in individuals that were rejected by two or three rejecters (Figure 2A, middle).

#### Rejecters vs. Innocent Individuals

There was no significant main effect of target, *F*(1, 10) = 2.39, *p* = 0.15, $\eta_{p}^{2}$ = 0.19 (*n* = 17), suggesting that the amount of money given to rejecters did not significantly differ from the amount given to innocent individuals (Table 2). In addition, there was no significant interaction between social anxiety and target, *F*(1, 10) = 2.14, *p* = 0.17, $\eta_{p}^{2}$ = 0.18. Although the data points in Figure 2A (right) are quite dispersed, it appears as if, socially anxious individuals gave more money to innocent individuals while reducing the amount for rejecters.

### Approach-Avoidance Task

#### Rejection vs. Acceptance

For AAT response toward human pictures, a non-significant trend of condition was found, *F*(1, 30) = 3.91, *p* = 0.06, $\eta_{p}^{2}$ *=* 0.12, with rejected individuals (*n* = 25) tending to have a more positive score than non-rejected individuals (*n* = 9). This means that they turned faces (pulled the joystick) quicker toward themselves (approach) than they turned them (pushed the joystick) away (avoidance; see Table 2). There was no significant interaction between social anxiety and condition, *F*(1, 30) = 2.53, *p =* 0.12, $\eta_{p}^{2}$ = 0.08. Visual inspection of Figure 2B (left) shows indeed that AAT scores in the rejection conditions seemed slightly higher than in the acceptance condition. With higher degrees of social anxiety, individuals seemed to show increasing approach when accepted, and more avoidance when rejected, although it is likely that that these differences may have been caused by some extreme values.

#### Number of Rejecters

A non-significant trend for number of rejecters was found, *F*(1, 26) = 2.82, *p =* 0.06, $\eta_{p}^{2}$ = 0.25. This seems to imply that, in general, non-rejected individuals tended to be quicker to turn faces away (avoidance), whereas individuals that were rejected by one or three individuals were quicker to turn faces towards (approach) themselves (see Table 2). In addition, a non-significant trend of an interaction with social anxiety was found, *F*(1, 26) = 2.85, *p =* 0.06, $\eta_{p}^{2}$ = 0.25. Exploratory correlations between social anxiety and AAT scores revealed that increasing degrees of social anxiety were related to more avoidance, but only after rejection by two others, *r* = −0.72, *p* = 0.03 (two-tailed). In all other rejection conditions, the correlations were not significant, with all *p*’s > 0.29. Visual inspection of Figure 2B (middle) suggests that the significantly steeper decline of the slope for two rejecters may be caused by an extreme observation in the data, while the slightly positive slope for one rejecter was probably caused by an extreme observation as well. Consequently, this could mean that an increasing number of rejecters may generally be related to increasing avoidance responding when degrees of social anxiety accumulate.

#### Rejecters vs. Innocent Individuals

Results revealed a non-significant trend of target, *F*(1, 13) = 4.00, *p* = 0.07, $\eta_{p}^{2}$ = 0.24. Participants (*n =* 17) had a negative AAT score/stronger avoidance-tendency toward their rejecters, while showing a (more) positive AAT score/approach-tendency toward innocent individuals (see Table 2). In addition, a non-significant trend of an interaction was found between social anxiety and target, *F*(1, 13) = 3.55, *p* = 0.08, $\eta_{p}^{2}$ = 0.21. Explorative, two-tailed correlation analyses; however, revealed no significant relationship between social anxiety and AAT scores neither in response to rejecters, *r* = 0.40, *p* = 0.13, nor in response to innocent individuals *r* = −0.41, *p* = 0.10. Visual inspection of Figure 2B (right) showed that an increasing degree of social anxiety seemed to be associated with an increase of approach tendencies toward rejecters and an increase of avoidance impulses toward accepters. Yet, it has to be acknowledged that the data points are quite scattered, deeming the statistical results to be inconclusive.

### Manipulation Check

#### Rejection vs. Acceptance

**Figure 3:**
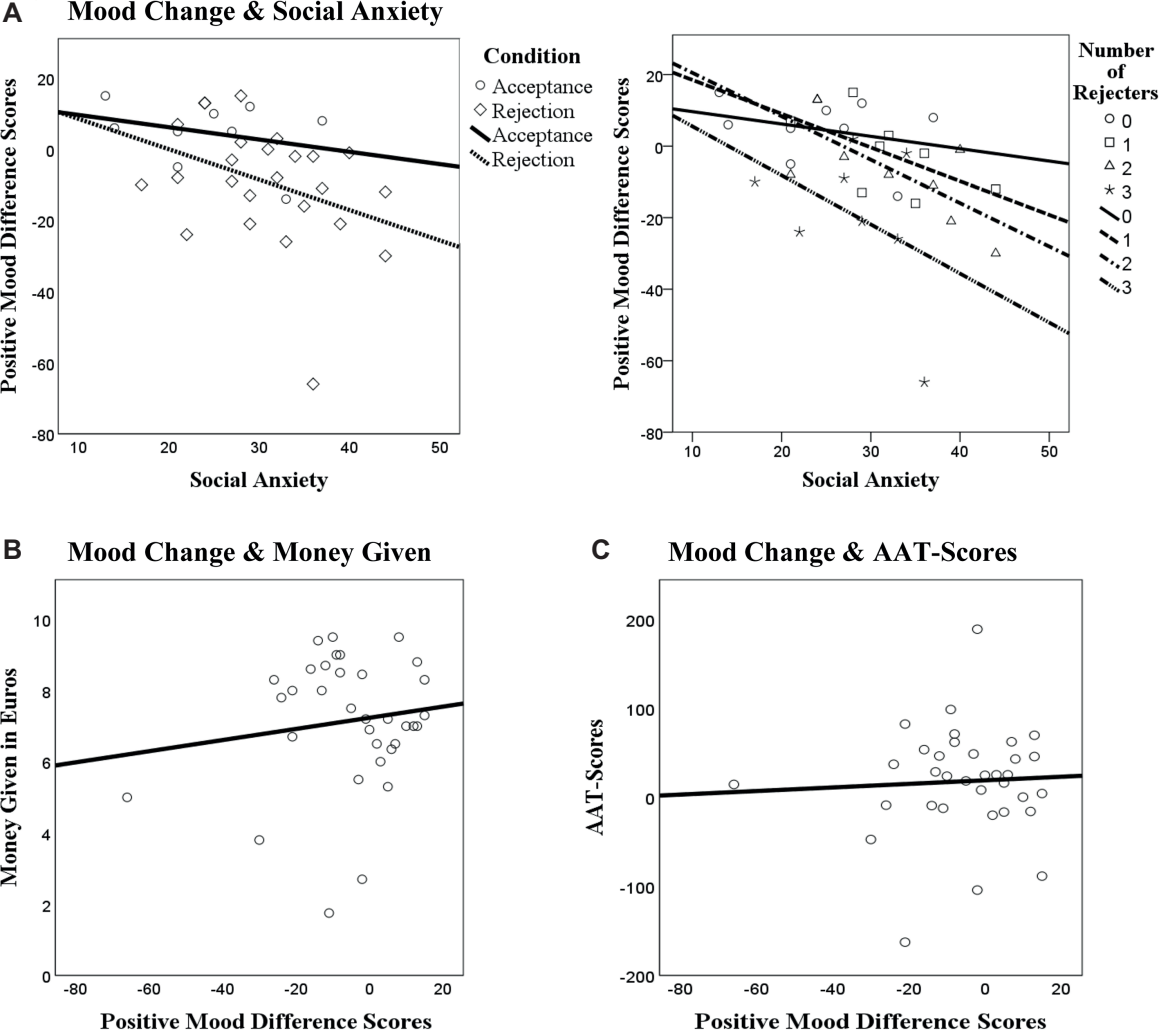
Scatterplots showing the relationship between changes in positive mood, social anxiety, and conditions **(A)** and the relationship between changes in positive mood and money given **(B)** as well as approach-avoidance scores **(C)**.

When looking at the different subjective mood reports, there was no main effect of time with regard to positive mood, *F*(1, 32) = 0.58, *p* = 0.45, $\eta_{p}^{2}$ = 0.02. However, a significant interaction was found between time and condition, *F*(1, 32) = 5.39, *p* = 0.03, $\eta_{p}^{2}$ = 0.14. Accepted individuals (*n =* 9) showed an increase in positive mood and rejected participants (*n* = 25) showing a decrease in positive mood. For feelings of anger, a non-significant trend of time was found *F* (1, 32) = 3.51, *p* = 0.07, $\eta_{p}^{2}$ = 0.10, showing an increase in anger from before to after the manipulation; however, no significant interaction was found with condition, *F*(1, 32) = 2.14, *p* = 0.15, $\eta_{p}^{2}$ = 0.06. No time effects were found for desire to escape, *F*(1, 32) = 1.86, *p* = 0.18, $\eta_{p}^{2}$ = 0.06, and anxiety, *F*(1, 32) = 1.65*, p* = 0.21, $\eta_{p}^{2}$ = 0.05, nor did significant interaction effects occur, *F*(1, 32) = 2.07, *p* = 0.16, $\eta_{p}^{2}$ = 0.06., *F*(1, 32) = 0.15, *p* = 0.70, $\eta_{p}^{2}$ = 0.01, respectively (Table 3). Visual inspection of Figure 3A (left) supports the idea that accepted participants showed an increase in mood and rejected participants, a decline. With regard to increasing degrees of social anxiety, the mood decline appeared be particularly strong.

**Table 3:** Affect-change scores across the varying rejection conditions.

	Number of rejecters							
	Zero (*n* = 9)		One (*n* = 8)		Two (*n* = 9)		Three (*n* = 8)	
Variable	*M*	SD	*M*	SD	*M*	SD	*M*	SD
Positive mood	4.67	8.97	−2.25	10.79	−6.22	14.10	−19.50	21.37
Desire to escape	−0.22	10.49	6.25	19.02	2.67	12.16	17.25	17.77
Anger	0.89	7.83	4.63	13.11	3.78	6.69	13.75	14.32
Anxiety	−5.56	18.64	1.13	6.67	−2.89	11.33	−7.25	26.76

#### Number of Rejecters

For changes in positive mood, a significant main effect of time was found, *F*(1, 30) = 5.52, *p* = 0.03, $\eta_{p}^{2}$ = 0.16, showing an overall decrease in positive mood from before to after the manipulation. In addition, there was a significant interaction with number of rejecters, *F*(1, 30) = 4.14, *p* = 0.01, $\eta_{p}^{2}$ = 0.29. The group with three rejecters had the strongest decrease in positive mood, followed by two rejecters and one rejecter, showing that the more individuals a participant was rejected by the stronger the decline in positive mood was. In contrast, the group with no rejecters showed an increase in positive mood (Table 3). For anger, a significant time effect was found, *F*(1, 30) = 9.67, *p* = 0.004, $\eta_{p}^{2}$ = 0.24, showing an overall increase in anger. For desire to escape a significant time effect was found as well, *F*(1, 30) = 6.28, *p* = 0.02, $\eta_{p}^{2}$ = 0.17, showing an overall increase of the desire to avoid from before to after the manipulation. However, for anxiety, no significant time effect was found, *F*(1, 30) = 1.48, *p* = 0.23, $\eta_{p}^{2}$ = 0.05. No further interaction effects were found between time and condition, neither for anger, desire to escape, nor anxiety, all *F’s* < 2.21, all *p*’s > 10 (see Table 3 for descriptives). Here, visual inspection (Figure 3A, right) supports the notion that mood declines differently when accepted and rejected conditions are compared and that social anxiety may amplify the drop in mood but that the degree of rejection does not seem to matter herein.

### Exploratory Analyses

#### Relationship Between Changes in Positive Mood and Social Anxiety

To explore the correlation between social anxiety and changes in subjective mood, difference scores of mood were calculated (Mood_After_ – Mood_Before_) and were subsequently entered in exploratory *post hoc* correlation analyses together with social anxiety scores. These correlation analyses (two-tailed) revealed that social anxiety was overall (*N =* 34) significantly negatively correlated to change in positive mood, *r* = −0.43, *p* = 0.01, meaning that the higher a person’s social anxiety was, the stronger was their decrease in positive mood. When exploring this relationship in more detail, it appeared that this relationship showed a non-significant trend for individuals that were rejected, *r* = −0.36, *p* = 0.08 (*n = 25*), but no significance for those being accepted, *r* = −0.31, *p =* 0.41 (*n =* 9). Visual inspection of Figure 3A (left) indeed shows a stronger decrease in mood with higher degrees of social anxiety both for accepted as well as rejected participants. However, the mood decline does appear to be stronger for rejected individuals. In addition, when looking at the specific number of rejecters, it was revealed that the relationship between changes in positive mood and social anxiety was only significant when participants were rejected by two individuals, *r* = −0.72, *p* = 0.03 (Figure 3A, right). The correlations for rejections by zero individuals (thus accepted), *r* = −0.31, by one, *r =* −0.59, and three individuals, *r* = −0.41 were not significant, all *p*’s > 0.10. Visual inspection of Figure 3A (right) on the other hand, does not provide clear-cut support for the idea that rejection by two rejecters should be significantly stronger related to social anxiety than rejection by one or three rejecters. In fact, the decline of the slopes seems fairly similar, with the regression lines of the conditions with two vs. three rejecters appearing almost parallel.

#### Relationship Between Changes in Positive Mood, Money Given and Approach-Avoidance Task

No significant relationship was found between changes in positive mood and amount of money given, *r* = 0.14, *p* = 0.43. For AAT responses, no significant relationship was found with changes in positive mood, *r* = 0.05, *p* = 0.76. Visual inspection of Figure 3 indeed shows a mostly linear slope for both, money given (Figure 3B) as well as AAT responses (Figure 3C). However, extreme values on the lower side of the mood changes may have influenced the slopes to some degree.

## Discussion

The current study proposed and piloted an experimental setup to explore explicit and implicit pro-social behavior following rejection in relation to social anxiety. In addition, the setup was used to examine whether this behavior varies with different degrees of rejection and toward whom the pro-social behavior is directed: the perpetrators of rejection or innocent individuals.

Overall, a non-significant trend as well as visual inspection of the data suggested that people gave less money when they were rejected and more when they were accepted. This seems contrary to our prediction based on the belongingness hypothesis by Baumeister and Leary (1995) and previous studies showing that participants responded in a compensatory pro-social manner after rejection (e.g., Williams et al., 2000; Lakin et al., 2008; Mallott et al., 2009). However, when taking social anxiety into account, a different pattern emerged. Results seem to indicate that people with higher levels of social anxiety (SA) gave less money when rejected while people with low levels of SA seemed to give more money. In the acceptance condition, the opposite pattern seems to emerge, although only from the visually plotted data not from the statistical analyses. Yet, these results correspond with our hypothesis as well as with research findings from Mallott et al. (2009) whom reported that low socially anxious participants displayed more pro-social behavior after rejection, whereas HSAs showed a trend toward less pro-social behavior. This hints at the possibility that the belongingness hypothesis does hold true but only for those with medium to low degrees of social anxiety. This idea also seems to be compatible with the rejection sensitivity model (Downey and Feldman, 1996), which posits that highly rejection sensitive individuals more readily perceive negativity and a high possibility of rejection in social situations, which can lead to maladaptive overreactions. For socially anxious individuals, this becomes even more pressing: Although HSAs’ core fear entails *expectation* of inevitable rejection, it seems as if they “secretly abandon” any thought about how to handle *true* rejection. This seems in line with cognitive models of SAD (e.g., Rapee and Heimberg, 1997) as well as research suggesting that HSAs may preferentially process belief-confirming above belief-disconfirming information (Belief-bias; Vroling et al., 2016). In addition, it may be the case that while HSAs have a whole repertoire of emotions and strategies available to try and manage their fears and prevent their utmost nightmare to become true, they do not know how to behave once rejection does indeed occur. Maladaptive overreactions in such a situation might be explained by the social skills deficit theory (for review, see Levitan and Nardi, 2009; for critical discussion, see also: Schneider and Turk, 2014) which suggests that people with social anxiety may have failed to learn effective social behavior by not fitting into environmental demands and experiencing extreme anxiety. Taking this theory into account, it may be the case that people with higher degrees of social anxiety might not have learned how to effectively deal with rejection and to regain acceptance by acting in a compensatory pro-social manner, primarily because they may have effectively avoided experiencing and dealing with true rejection.

Unlike expected, no significant differences were found in amount of money given when someone was rejected by one, two, or three people nor was it found to be related to social anxiety. This is particularly surprising when considering that the more individuals a participant was rejected by, the stronger their decline was in terms of positive mood. While the latter seems to fit with findings that an increasing level of ostracism was associated with an increasing impact on participants’ emotions and mood (Sandstrom et al., 2017; Tobin et al., 2018; but also, Pfeiffer and In-Albon 2016), the fact that these studies have not looked at the association between subjective self-report measures and actual behavior makes it difficult to draw solid solutions. It is possible that a stepwise decline of, e.g., positive mood does not directly translate to distinguishable differences in overt behavior. The rating of creativity and the consequential allotting of money may be too much of an abstract conscious act to be driven by fine-grained mood differences triggered by rejection by, e.g., three rather than two others. When looking at the visual representation of the data, it is thinkable that the current results are driven by some extreme values and that an increase in social anxiety may after all have a distinct effect depending on the number of rejecters. In any case, to explore the idea of proportional transfer from emotion and mood to explicit behavior in more detail, the difference in number of rejecters, may need to be bigger (e.g., zero, three, six, and nine rejecters). In the same vein, it would be worthwhile to explore the idea of repeated and/or increasing degrees of rejection rather than having a one-shot setup (Buckley et al., 2004). In the present study, participants were only rejected once.

Contrary to our expectation, neither the statistical results nor visual inspection of the data pointed clearly at significant differences between rewards allotted to innocent individuals or specific perpetrators of rejection nor was there an influence of social anxiety. While on the one hand, rejected people, in general, have been found to indeed retaliate against their perpetrators when possible (e.g., Twenge and Campbell, 2003; Buckley et al., 2004); research suggests that socially anxious individuals should be even more inclined to do so (Ayduk et al., 1999, 2008). On the other hand, they tend to overgeneralize from one instance of rejection to subsequent situations and are consequently thought to be less accurate in distinguishing accepter from rejecter (Maner et al., 2007; Mallott et al., 2009; DeWall, 2013). Our current results seem to suggest, however, that compensatory responses after rejection might be more generalized and not specifically directed at certain individuals, irrespective of a person’s level of social anxiety. As stated earlier, the association between the specific cause of one’s negative mood and the act of allotting money based on the other’s creativity may be too weak to have a significant impact. In addition, there may be an important conceptual difference between retaliating against someone (Twenge and Campbell, 2003) and not rewarding someone, as was the case in our task. As a matter of fact, less reward can only be seen as punishment if the receiver has a comparison standard. A real punishment would be to allot no money at all for the rejecter(s), but here feelings of fairness (Arkin et al., 1980) or social desirability for which socially anxious individuals are very susceptible (Tanaka Matsumi and Kameoka, 1986) may take over. In future research, an option to truly retaliate against the other should be added to be able to contrast participants’ response to their responses when “rewarding” their rejecters.

With regard to implicit behavior, our results partly confirm our hypotheses: In general, rejected participants had a stronger approach tendency when compared to non-rejected participants, although visual data inspection suggests that a few extreme values may have been responsible. Social anxiety, however, did not play a significant role. Should the main effect be valid, it would be in line with the belongingness hypothesis (Baumeister and Leary, 1995). Again, the stronger approach tendency in rejected participants may indicate implicit compensatory behavior and the desire to reconnect. In the same line, research indicates that, after social exclusion, participants tended to focus more on pictures of positive faces Chen et al. (2017) and showed increased facial mimicry and emotion encoding accuracy (Cheung et al., 2015) propagating social approach. The lacking influence of social anxiety is surprising. Although not concerning punishment one would have expected that in line with Ayduk et al. (1999, 2008) individuals with high degrees of SA would be more inclined to avoid after rejection, contrasting with the general social approach tendencies of non-anxious individuals. This is difficult to explain, but the small sample size may have played a role in here. Having a look at the differentiation between the different number of rejecters may help to shed light on the matter.

Non-rejected individuals showed an overall tendency to have more negative scores than those that were rejected by one or more individuals, again, seemingly indicating a stronger approach tendency in rejected individuals. In addition, a trend of an interaction between number of rejecters and social anxiety was found. Further analyses revealed that the higher one’s social anxiety was, the more negative their AAT scores were, indicating a stronger avoidance tendency. This relationship, however, was revealed only in participants that were rejected by two individuals. When inspecting the data visually, it seems as if a few extreme observations may have influenced the results here. When disregarding them, it appears as if the slope of avoidance per number of rejecter may have declined steeper per added rejecter and increasing social anxiety. Yet, in the light of the small sample size, this is quite speculative and cannot be tested statistically. Interestingly, however, exploratory analyses have also revealed a significant relationship between change in positive mood and social anxiety in this particular condition, showing a higher decrease in mood in those scoring higher on anxiety. Again, this relationship may be inflated by extreme observations. Yet, these results seem to be partly in line with expectations, as it was believed that those high in social anxiety would be more negatively affected and that they would show a stronger avoidance tendency in response to rejection in comparison to those low in social anxiety due to their sensitivity to socially stressful situations (Downey and Feldman, 1996; Rapee and Heimberg, 1997). When disregarding the possibility that small sample size and extreme observations may have been skewing the results, it is puzzling that this relationship would be strongest when rejected by two individuals. One possible explanation could be the ambiguity of such a situation. When one is rejected by only one out of three participants, it could be rationalized that this individual was merely an exception. In the case of three rejecters, it might be the most painful (as the results of our manipulation check also suggest), but the verdict is at least unanimous. Previous literature indeed suggests that socially anxious individuals seem to interpret and respond to ambiguous situations in a more negative way (Stopa and Clark, 2000; Zadro et al., 2006; Staugaard, 2010). However, a study by Zimmer-Gembeck and Nesdale (2013) found that highly rejection sensitive participants were more likely to report retribution as their anticipated response to ambiguous situations and more likely to report avoidance as their anticipated response to non-ambiguous situations. These results do not seem to correspond with ours, as avoidance seems more similar to withdrawal then to retribution. The difference in methodology calls into question whether the same results can be expected. Yet, it does indicate that the importance of ambiguity when studying responses to rejection needs to be taken into account although statistical restrictions of our own results should kept in mind.

When looking at differences in automatic responses directed toward pictures of rejecters and accepters or innocent others, it was found that in general participants had a stronger avoidance tendency towards their rejecters. However, when taking social anxiety in account, it was revealed that this was mostly the case for people with low degrees of SA. With increasing levels of SA, there was a stronger approach tendency toward rejecters and a stronger avoidance tendency toward innocent individuals. This seems contrary to our expectation based on previous literature (Maner et al., 2007; Mallott et al., 2009; DeWall, 2013), suggesting that the distinction between specific sources of rejection and innocent individuals would diminish with higher degrees of SA. Furthermore, this differentiation in responses was not found in our reward task. This may mean that explicit compensatory responses after rejection might be more generalized since they are diluted by cognitive elaboration and social desirability, implicit approach or avoidance tendencies are more of an impulsive evaluative nature bypassing conscious exploration. Intriguingly, it seems that HSA individuals may not tend to show overt re-affiliate behavior after being rejected and make no distinction between rejecters and acceptors, but they may still feel inclined to re-affiliate on an implicit level particularly toward former rejecters. This discrepancy is difficult to disentangle, at this point, but it clearly indicates that HSAs show explicit and implicit behaviors after acceptance as well as after rejection that are opposite to what would be normally expected thereby exacerbating the chances of acceptance. However, the underlying mechanisms warrant further investigation.

In order to explore indirect effects of mood changes on the dependent measures, additional analyses revealed that the higher a rejected participant’s social anxiety was, the stronger their decrease of positive mood was. This makes perfect sense when keeping in mind that rejection is the core fear of HSAs. However, this decrease of positive mood did not directly relate to either the explicit (giving money) nor the implicit (AAT scores) responses towards rejection. Instead, it seems that social anxiety by itself plays an important role in how someone emotionally responds to rejection, while this emotional change does not seem to explain any additional variance in the measured behaviors. This is partially in line with Heeren et al. (2017) suggesting that differences in socially anxious responding after rejection may show not so much in direct responding but rather in delayed recovery (see also, Zadro et al., 2006).

Apart from these theoretical considerations, a number of limitations have to be acknowledged. First and most importantly, the sample size of this pilot study (*N* = 34) was very small, especially considering the number of groups (with different sample sizes) which were compared, resulting in an overall low statistical power and some of the investigated effects *approaching*, rather than reaching significance. In addition, comparing, e.g., 9 (accepted) with 25 (partially) rejected participants is critical as is conducting correlational analyses in the subsamples with *n*’s of 8 and 9. This restricts the explanatory power of our results considerably. Although bootstrapping was applied to simulate a larger sample sizes, and visual inspection of the data was used to identify possible flaws in the validity of the statistical results, it would have been more ideal to have a larger group of participants available. The found effect sizes were fairly large, around $\eta_{p}^{2}$ *=* 0.12 (which translates to Cohen’s *d =* 0.74, Cohen, 1988) which suggests that our found results may still be valuable. However, as our sample size was small, it also increases the likelihood of sampling bias with an overestimation of these effects. Although it was our intention to support the workability of our suggested experimental procedure by pilot data first, an extensive replication with a larger sample size is nevertheless highly recommended to confirm the reported results. Yet, we are confident that the data has potential to show the relevance of varying numbers the of rejecters systematically and the target of re-affiliative pro-social behaviors.

In addition, expected distinctions may not have shown up in the AAT as the number of pictures that were included of unknown innocent individuals was overwhelmingly larger than the amount of pictures of individuals that the participants had encountered previously. In general, the large number of trials may eventually have led to trained rather than impulsive responding in the end. In future research, it is therefore recommended to consider to either adjust the number of pictures and reduce the number of trials.

Furthermore, while the predicted drop in positive mood dependent on the number of rejecters makes us quite confident that our manipulation worked, we need to acknowledge that the other affect states, anger, anxiety, and desire to escape were left unaffected by the manipulation. Merely anger underwent a general increase from pre- to post-manipulation but was irrespective of whether participants were rejected or accepted. Especially, the lack of effect on anger in response to rejection seems to contradict previous studies: Anger is one of the foremost feelings found to be evoked by social exclusion, which leads in turn to increases in aggressive tendencies (e.g., Williams, 2009). However, the temporal need-threat model by Williams (2009) also suggests that, following exclusion, individuals will always display re-inclusion facilitating behavior, except when re-inclusion seems unlikely. In that case, anti-social responses, such as aggression, are used to regain control or to force others to recognize their existence (Gerber and Wheeler, 2009). In our study, even though participants did not get a chance to interact face-to-face with their rejecters, they may have regarded the reward paradigm as a re-inclusion opportunity, and may therefore not have felt this need. In addition, feelings of anxiety or desire to escape may not have been significantly affected as participants no longer expected a face-to-face interaction, thereby making the situation less stressful.

Lastly, the current sample only included participants with social anxiety at a sub-clinical level; therefore, it cannot be said with certainty that these results are generalizable to the clinical population. Yet, Rapee and Heimberg (1997) do suggest that social anxiety is distributed along a continuum and effects at the high end should be quantitatively but not qualitatively different in clinical as compared to subclinical samples. Consequently, effects found here should be more pronounced in clinical samples.

Keeping primarily the statistical restrictions but also the other limitations in mind, it was nevertheless demonstrated that our proposed experimental set-up is a promising way to explore several aspects related to explicit as well as implicit behavior in response to social rejection. The results from this pilot study suggest that outright social rejection, as well as its degree of severity, may have significant effects on an individual’s emotional state and behavioral responding. After outright rejection, people tend to display explicit compensatory actions but only those with low degrees of social anxiety. An increasing degree of social anxiety, generally reverses these pro-social patterns: allotting less reward and showing more avoidance tendencies after rejection. With regard to the distinction between the target of the pro-social behaviors, rejecters, and accepters, only implicit behaviors tendentiously showed a difference: Avoidance of the first and approach of the latter. Again, social anxiety changes this pattern, showing a stronger approach tendency towards rejecters. In sum, it seems that, by behaving incompatible to what is typically expected after assumed as well as true rejection, individuals suffering from social fears may actually precipitate the situation they fear the most. Future research would need to seek out whether a lack of skill or the anxiety itself drives these behaviors. Regarding the methods, the presented paradigm is promising for future research as manipulating degrees of overt rejection eliminates interpretations of ambiguous ostracism manipulations and distils responses to outright and unmistakable rejection. In addition, it opens avenues to measure explicit and implicit behavioral responses toward increasing numbers of “rejecters” as compared to “accepters” and other unknown innocent others. Our results in addition to the fact that past rejections seem to increase social anxiety in future interactions and rejections (Levinson et al., 2013; Stenseng et al., 2014) could also inspire adjustments of treatment regiments for social anxiety disorder specifically aiming at behavioral responses after *outright* rejection in addition to (cognitions about) *putative* rejection. Providing training in adequate behavior in rejection, as well as acceptance situations, would be highly valuable especially for socially anxious individuals as such situations are ubiquitous in everyday life.

## Ethics Statement

This study was carried out in accordance with the recommendations of the Ethical Committee of the Faculty of Social Sciences, Radboud University Nijmegen, the Netherlands (ECSS), with written informed consent from all subjects. All subjects gave written informed consent in accordance with the Declaration of Helsinki. The protocol was approved by the ECSS (Decision number: ECSW-2018-036).

## Author Contributions

While the initial research idea was set out by W-GL, both authors contributed equally to conception and design of the study. JW conducted the study, performed the statistical analysis, and wrote the first draft of the manuscript. W-GL supervised the whole project and (re-)wrote sections of the manuscript. All authors contributed to manuscript revision, read and approved the submitted version.

### Conflict of Interest Statement

The authors declare that the research was conducted in the absence of any commercial or financial relationships that could be construed as a potential conflict of interest.
